# Supplementary material for: A Minimal Genetic Passkey to Unlock Many Legume Doors to Root Nodulation by Rhizobia
Source: Genes (Basel). 2020 May 7;11(5):521. doi: 10.3390/genes11050521 (PMC7290934; doi:10.3390/genes11050521)
Supplement: Supplementary file 1 [file genes-11-00521-s001.pdf]

SUPPLEMENTARY DATA TO

"A minimal genetic passkey to unlock many legume doors to root nodulation by rhizobia"

by Jovelyn Unay and Xavier Perret

University of Geneva, Department of Botany and Plant Biology, Sciences III, 30 quai Ernest-Ansermet, CH-1211 Geneva, Switzerland

Correspondence to: Xavier.Perret@unige.ch

Table S1. Strains and plasmids used in this study.

Strain	Characteristics	Source / Reference
ANU265	Derivative of NGR234 cured of its symbiotic plasmid pNGR234a, Sp^R	Morrison et al. (1983)
ANU265::pMiniSym1	ANU265 transconjugant carrying pMiniSym1, Sp^R, Km^R	This work
ANU265::pMiniSym2	ANU265 transconjugant carrying pMiniSym2, Sp^R, Km^R	This work
ANU265::pMiniSym2-Gus	ANU265 transconjugant carrying pMiniSym2-Gus, Sp^R, Km^R	This work
CBM832	*Cupriavidus taiwanensis* strain that fixes nitrogen with *Mimosa pudica*	Marchetti et al. (2010)
CMG6	Proficient *Mesorhizobium ciceri* strain isolated from *Cicer arietinum*	Ben Romdhane et al. (2007)
E. coli DH5α	F- Φ80*lac*ZΔM15, Δ(*lac*ZYA-*arg*F), *rec*A1, *end*A1, *hsd*R17, *pho*A, *sup*E44, *gyr*A96 *rel*A1	Woodcock et al. (1989)
E. coli NEB 10-beta	DH10B derivative for cloning large plasmids, *endA1-*	NEB (Ipswich, MA, USA)
MAFF303099	Strain of *Mesorhizobium japonicum* that is proficient on several *Lotus* spp.	Kaneko et al. (2000)
NGR234	Rif^R-derivative of the *S. fredii* strain isolated from *Lablab purpureus* by M.J. Trinick	Stanley et al. (1988)
NGR234::pXPrpsL426	NGR234 transconjugant carrying pXPrpsL426, Rif^R, Sp^R	Fumeaux et al. (2011)
Sm1021	*Sinorhizobium meliloti* strain 1021 proficient on *Medicago sativa* cv. Gemini	Capela et al. (2001)
WSM419	*Sinorhizobium medicae* strain that fixes nitrogen with *M. truncatula* cv. Jemalong	Reeve et al. (2010)
**Plasmid**	**Characteristics**	**Source / Reference**
pBluescript II KS+ (pKS)	ColEI-based phagemid, *lacαZ*+, Ap^R	Stratagene
pHP45 Ω Km	pHP45 carrying the kanamycin resistant (Km^R) Omega interposon	Fellay et al. (1987)
pMiniSym1	7,282 bp RK2-based, mobilisable, low copy number vector for mini-symbiotic plasmids	This work
pMiniSym2	pMiniSym1 with *nodD1* and *nodABCIJS* genes/promoters of NGR234, Km^R	This work
pMiniSym2-Gus	pMiniSym2 with GusA constitutive expression, Km^R	This work
pKS-PrpsL-GUS-1	pKS with a 2,313 bp *Pst*I-*Sac*I fragment with the *uidA* gene and *rpsL* promoter of pXPrpsL426, Ap^R	This work

Table S1. Continuation

Plasmid	Characteristics	Source / Reference
pNB3-nodD1-10	pKS with NGR234 *nodD1* and native promoter as 1,347 bp *Spe*I fragment, Ap^R	This work
pNB8-nodABCIJ-30	pKS with NGR234 *nodABCIJ* and native promoter as 4,996 bp *Spe*I fragment, Ap^R	This work
pNB12-nodS-4	pKS with NGR234 *nodS* and native promoter as 1,224 bp *Spe*I fragment, Ap^R	This work
pRK2oriT-1	pKS with pRK7813 origin of transfer (*oriT*) as 410 bp *Spe*I fragment, Ap^R	This work
pRK2oriV-1	pKS with pRK7813 origin of replication (*oriV*) as 854 bp *Spe*I fragment, Ap^R	This work
pRK2trfA-7	pKS with pRK7813 *trfA* and native promoter as 1,782 bp *Kpn*I fragment, Ap^R	This work
pRK2013	Tra^+ helper plasmid for tri-parental mobilisation, Km^R	Figurski and Helinski (1979)
pRK7813	Broad host-range IncP1 and RK2 based costramid, Tc^R	Jones and Gutterson (1987)
pSpsAB-4	pKS with NGR234 *spsAB* and native promoter as 2,374 bp *Spe*I fragment, Ap^R	This work
pXB182	Lorist2 based cosmid covering a section of pNGR234a with the *nodABCIJ* genes, Km^R	Perret et al. (1991)
pXB1027	Lorist2 based cosmid covering a section of pNGR234a with the *nodSU* genes, Km^R	Perret et al. (1991)
pXB1357	Lorist2 based cosmid covering a section of pNGR234a with the *nodD1* gene, Km^R	Perret et al. (1991)
pXPrpsL426	pRG960 with *rpsL* promoter of NGR234 driving constitutive expression of *uidA*, Sp^R	Fumeaux et al. (2011)

References

Ben Romdhane, S.; Tajini, F.; Trabelsi, M.; Aouani, M.E.; Mhamdi, R. Competition for nodule formation between introduced strains of *Mesorhizobium ciceri* and the native populations of rhizobia nodulating chickpea (*Cicer arietinum*) in Tunisia. *World J. Microbiol. Biotechnol.* **2007**, *23*, 1195-1201, doi:10.1007/s11274-006-9325-z.

Capela, D.; Barloy-Hubler, F.; Gouzy, J.; Bothe, G.; Ampe, F.; Batut, J.; Boistard, P.; Becker, A.; Boutry, M.; Cadieu, E., et al. Analysis of the chromosome sequence of the legume symbiont *Sinorhizobium meliloti* strain 1021. *Proc. Natl. Acad. Sci. USA* **2001**, *98*, 9877-9882, doi:10.1073/pnas.161294398.

Fellay, R.; Frey, J.; Krisch, H. Interposon mutagenesis of soil and water bacteria: a family of DNA fragments designed for in vitro insertional mutagenesis of Gram-negative bacteria. *Gene* **1987**, *52*, 147-154, doi:10.1016/0378-1119(87)90041-2.

Figurski, D.H.; Helinski, D.R. Replication of an origin-containing derivative of plasmid RK2 dependent on a plasmid function provided in trans. *Proc. Natl. Acad. Sci. USA* **1979**, *76*, 1648-1652, doi:10.1073/pnas.76.4.1648.

Fumeaux, C.; Bakkou, N.; Kopcińska, J.; Golinowski, W.; Westenberg, D.J.; Müller, P.; Perret, X. Functional analysis of the *nifQdctA1*y4vGHIJ operon of *Sinorhizobium fredii* strain NGR234 using a transposon with a NifA-dependent read-out promoter. *Microbiol.* **2011**, *157*, 2745-2758, doi:10.1099/mic.0.049999-0.

Jones, J.D.; Gutterson, N. An efficient mobilizable cosmid vector, pRK7813, and its use in a rapid method for marker exchange in *Pseudomonas fluorescens* strain HV37a. *Gene* **1987**, *61*, 299-306, doi:10.1016/0378-1119(87)90193-4.

Kaneko, T.; Nakamura, Y.; Sato, S.; Asamizu, E.; Kato, T.; Sasamoto, S.; Watanabe, A.; Idesawa, K.; Ishikawa, A.; Kawashima, K., et al. Complete genome structure of the nitrogen-fixing symbiotic bacterium *Mesorhizobium loti*. *DNA Res.* **2000**, *7*, 331-338, doi:10.1093/dnares/7.6.331.

Marchetti, M.; Capela, D.; Glew, M.; Cruveiller, S.; Chane-Woon-Ming, B.; Gris, C.; Timmers, T.; Poinsot, V.; Gilbert, L.B.; Heeb, P., et al. Experimental evolution of a plant pathogen into a legume symbiont. *PLoS Biol.* **2010**, *8*, e1000280, doi:10.1371/journal.pbio.1000280.

Morrison, N.A.; Hau, C.Y.; Trinick, M.J.; Shine, J.; Rolfe, B.G. Heat curing of a Sym plasmid in a fast-growing *Rhizobium* sp. that is able to nodulate legumes and the nonlegume *Parasponia* sp. *J. Bacteriol.* **1983**, *153*, 527-531.

Perret, X.; Broughton, W.J.; Brenner, S. Canonical ordered cosmid library of the symbiotic plasmid of *Rhizobium* species NGR234. *Proc. Natl. Acad. Sci. USA* **1991**, *88*, 1923-1927, doi:10.1073/pnas.88.5.1923.

Reeve, W.; Chain, P.; O'Hara, G.; Ardley, J.; Nandesena, K.; Bräu, L.; Tiwari, R.; Malfatti, S.; Kiss, H.; Lapidus, A., et al. Complete genome sequence of the *Medicago* microsymbiont *Ensifer* (*Sinorhizobium*) *medicae* strain WSM419. *Stand. Genomic Sci.* **2010**, *2*, 77-86, doi:10.4056/sigs.43526.

Stanley, J.; van Slooten, J.; Dowling, D.N.; Finan, T.; Broughton, W.J. Molecular cloning of the *ntrA* gene of the broad host-range *Rhizobium* sp. NGR234, and phenotypes of a site-directed mutant. *Mol. Gen. Genet.* **1989**, *217*, 528-532, doi:10.1007/BF02464927.

Woodcock, D.M.; Crowther, P.J.; Doherty, J.; Jefferson, S.; DeCruz, E.; Noyer-Weidner, M.; Smith, S.S.; Michael, M.Z.; Graham, M.W. Quantitative evaluation of *Escherichia coli* host strains for tolerance to cytosine methylation in plasmid and phage recombinants. *Nucleic Acids Res.* **1989**, *17*, 3469-3478, doi:10.1093/nar/17.9.3469.

Table S2. Oligonucleotides and templates for PCR amplification and cloning of gene blocks in separate constructs.

The *Spe*I (5'-A'CTAGT-3') and *Kpn*I (5'-GGTAC'C-3') restriction sites used for cloning amplicons are underlined.

**Target**	**Primer**	**Sequence** (5′ to 3′)	**Template**	**Cloned as** (bp)	**In construct**
NB8 + *nodABCIJ*	NB8-nodABCIJ-For	gggactagtagcggtattagc	pXB182	4,996	pNB8-nodABCIJ-30
	NB8-nodABCIJ-Rev	gtaactagtaggccatgtgctc			
NB12 + *nodS*	NB12-nodS-For	gaactagtctccatcacatccacc	pXB1027	1,224	pNB12-nodS-4
	NB12-nodS-Rev	cgactagttattccgcttctcc			
nodD1+ NB3	nodD1-NB3-For	gaactagtgctgcgcataggc	pXB1357	1,347	pNB3-nodD1-10
	nodD1-NB3-Rev	ttactagtggcaaggctgttgc			
PrpsL + *uidA*	PrpsL-GUS-For	gaaactagtatgaccatgattacg	pKS-PrpsL-GUS-1	2,594	pMiniSym2-Gus
	PrpsL-GUS-Rev	caactagtgggaagggcgatc			
oriV	RK2oriV-For	caactagtcgctgaatgt	pRK7813	854	pRK2oriV-1
	RK2oriV-Rev	caactagtcagtgagcgagg			
oriT	RK2oriT-For	cccttactagttggcttgg	pRK7813	410	pRK2oriT-1
	RK2oriT-Rev	ggactagtaagataccaggcg			
spsAB	spsAB-For	ctactagtcctggacttcgtcg	NGR234 gDNA	2,374	pSpsAB-4
	spsAB-Rev	gcactagtgggtggctatagg			
trfA	trfA-For	gtgggtaccgagcgatactga	pRK7813	1,782	pRK2trfA-7
	trfA-Rev	ggtggtacccagcggaagc			

Table S3. Primers and templates for amplifying gene blocks for Gibson assemblies and resulting constructs, with nucleotides matching templates underlined.

Gene block	Primer	Sequence (5′ to 3′)	Template	Construct
Omega-Km	GB-OmegaKm-For	tcaaggcgttagcccatccatggggttcgatacgttagcg	pHP45 Ω Km	pMiniSym1
	GB-OmegaKm-Rev	gcttcgctcagtatcgctcggcgacgttggtgaccttgatcc		
oriT	GB-OriT-For	gtttaaacactagtcctaggggcgcgcccatccgcttgccctcatctg	pRK2oriT-1	pMiniSym1
	GB-OriT-Rev	gcagtaacgggatgggcgatggtgtatccaacggcgtcagc		
oriV	GB-OriV-For	gctgacgccgttggatacaccatcgcccatcccgttactgc	pRK2oriV-1	pMiniSym1
	GB-OriV-Rev	cgatggaacgggtgcgtgaccagtgagcgaggaagcggaag		
spsAB	GB-spsAB-For	cttccgcttcctcgctcactggtcacgcacccgttccatcg	pSpsAB-4	pMiniSym1
	GB-spsAB-Rev	cgctaacgtatcgaaccccatggatgggctaacgccttga		
trfA	GB-trfA-For	gggcgcgcccctaggactagtgtttaaaccccctcgcacctttggtcgc	pRK2trfA-7	pMiniSym1
	GB-trfA-Rev	ggatcaaggtcaccaacgtcgccgagcgatactgagcgaagc		
nodD1	GB2-nodD1-For	cgctaacgtatcgaaccccagacgaaatggcaggagcggt	pNB3-nodD1-10	pMiniSym2
	GB2-nodD1-Rev	agcaggagaagcggaataacaatggtcggagtgtggcatg		
nodS	GB2-nodS-For	ttcagtcggagcacatggcctgtgatttcgacatggatgc	pNB12-nodS-4	pMiniSym2
	GB2-nodS-Rev	catgccacactccgaccattgttattccgcttctcctgct		
nodABCIJ	GB2-nodABCIJ-For	tcaaggcgttagcccatccagcggtattagcttcattgcc	pNB8-nodABCIJ-30	pMiniSym2
	GB2-nodABCIJ-Rev	gcatccatgtcgaaatcacaggccatgtgctccgactgaa		
pMiniSym1	GB2-OmegaKm-For	accgctcctgccatttcgtctggggttcgatacgttagcg	pMiniSym1	pMiniSym2
	GB2-spsAB-Rev	ggcaatgaagctaataccgctggatgggctaacgccttga		

Table S4. Primers used in qPCR to determine pMiniSym2 and pNGR234a copy numbers in ANU265 and NGR234, respectively.

**Primer**	**Sequence** (5′ to 3′)	**Gene**	**Replicon**	**Amplicon** (bp)
rpoC-F	GAGAAGTGCGGTGTCGAAGT	*rpoc*	chromosome	98
rpoC-R	TTCAGGAACCAGATGTGGGC			
rpsL-F	GAACTCGGCTCTGCGTAAGG	*rpsL*	chromosome	98
rpsL-R	AGTGCTCCTGAAGGTTGTGG			
nodB-For	TTTGACGACGGTCCTAACCC	*nodB*	pMiniSym2/pNGR234a	100
nodB-Rev	TAGCATAAGCCCCGATGACG			
nodD1-F	GAACAGCTTCGCTCTGGGA	*nodD1*	pMiniSym2/pNGR234a	100
nodD1-R	TTCCGATGAGCCAGATGAGC			

Table S5. Comparing the copy numbers of pNGR234a and pMiniSym2 plasmids.

The plasmid copy number (PCN) was obtained using the following formula $2^{[Ct(Chr)-Ct(plas)]}$ where the averaged Ct's for the chromosome (Chr) and for plasmid (plas) were obtained by combining Ct values for the chromosomal *rpoC* and *rpsL* genes and plasmid *nodB* and *nodD1* genes, respectively. Genomic DNA (gDNA) was extracted from NGR234 and ANU265::pMiniSym2 cells harvested at OD_{600} 0.8 and grown in either RMS or TY liquid media, using 50 µg/ml kanamycin (ANU265::pMiniSym2) or rifampicin (NGR234). For each gene, qPCR was performed in triplicate and using five gDNA concentrations (from 0.156 to 40 ng) as template. Primer sequences used for amplifying the *rpoC*, *rpsL*, *nodB* and *nodD1* target genes are listed in Table S4.

			Chromosome		Plasmid		
Strain	Medium	gDNA (ng)	Ct(Chr)	StDev [Ct(Chr)]	Ct(plas)	StDev [Ct(plas)]	PCN
NGR234	RMS	40	15.72	0.25	15.30	0.40	1.34
		10	17.39	0.05	17.11	0.38	1.22
		2.5	19.35	0.26	18.79	0.37	1.48
		0.625	21.65	0.37	21.21	0.42	1.36
		0.156	23.63	0.38	23.14	0.42	1.41
	TY	40	17.88	0.09	17.59	0.35	1.22
		10	19.97	0.19	19.63	0.32	1.27
		2.5	21.76	0.19	21.45	0.39	1.23
		0.625	23.88	0.34	23.72	0.42	1.12
		0.156	25.88	0.39	25.35	0.37	1.44
ANU265::pMiniSym2	RMS	40	16.47	0.07	16.49	0.31	0.99
		10	18.58	0.24	18.48	0.37	1.07
		2.5	20.73	0.34	20.41	0.32	1.25
		0.625	22.61	0.31	22.42	0.35	1.14
		0.156	24.74	0.31	24.34	0.30	1.32
	TY	40	17.36	0.08	16.51	0.15	1.80
		10	19.23	0.25	18.28	0.13	1.94
		2.5	21.18	0.34	20.20	0.09	1.97
		0.625	23.36	0.43	22.30	0.08	2.08
		0.156	25.27	0.37	24.32	0.09	1.94

Figure S1. pMiniSym2 confers to ANU265 a robust nodulation capacity on cowpea, siratro and *Leucaena leucocephala*. Photographs of shoots and corresponding roots of *Vigna unguiculata* cv. Red Caloona **(a)**, *Macroptilium atropurpureum* cv. Siratro **(b)** and *L. leucocephala* **(c)** plants that were harvested 28 days post-inoculation with NGR234, ANU265::pMiniSym2 or ANU265. Scale bars, 1 cm.

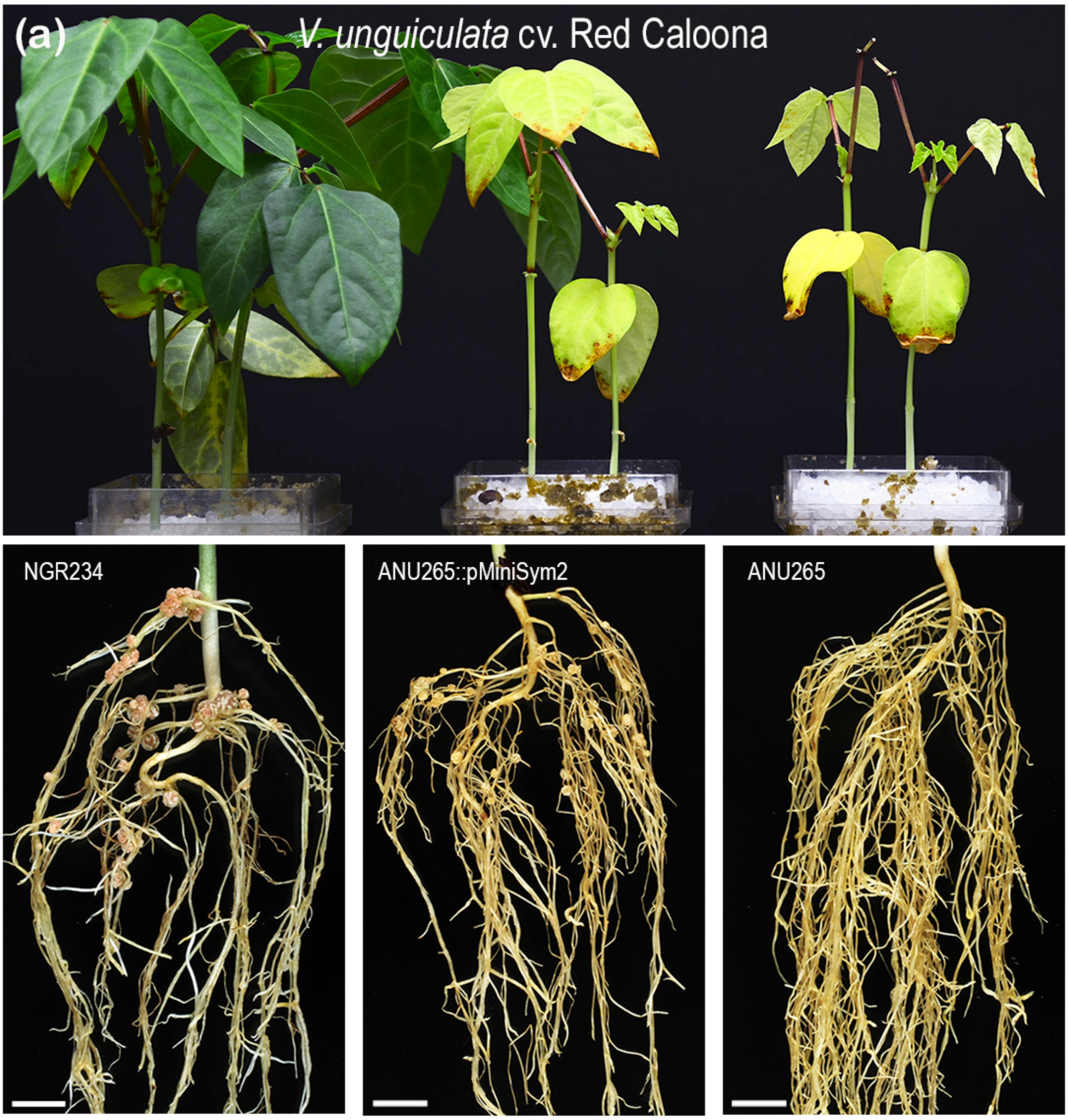

Figure S1 continues on next page

Figure S1. Continuation

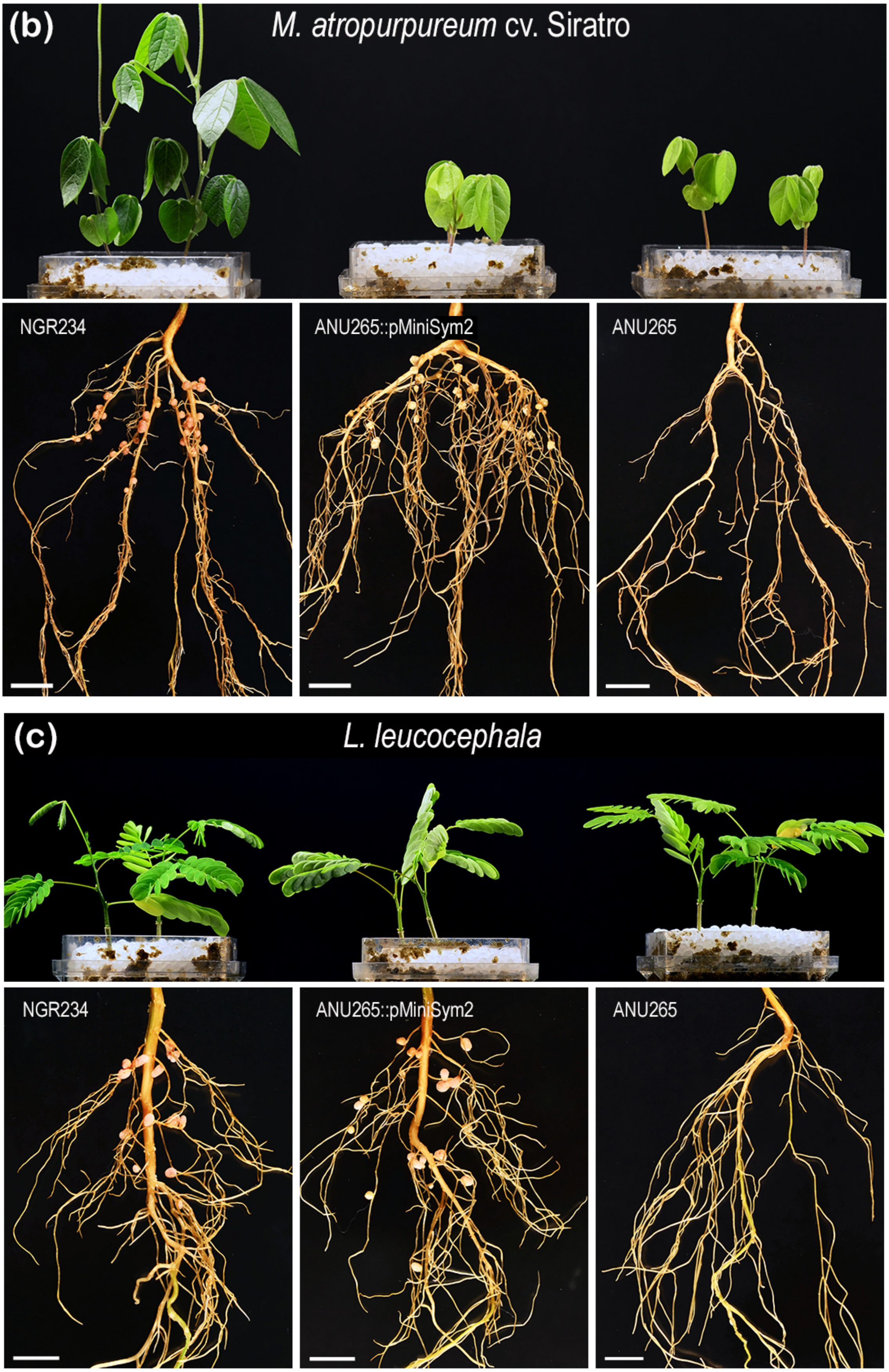

Figure S2. Capacity of ANU265::pMiniSym2-Gus to colonise root nodules is host-dependent. Sections of nodules formed on roots of *Vigna unguiculata* cv. Red Caloona, *Macroptilium atropurpureum* cv. Siratro and *Leucaena leucocephala* 28 days post-inoculation with ANU265::pMiniSym2-Gus or NGR234::pXPrpsL426 transconjugant strains. Nodule sections were photographed after staining for β-glucuronidase activity. More than a dozen nodules per inoculum and from several roots were examined per treatment. White circles delimit patches of Gus-stained cells occasionally observed in cowpea nodules formed by ANU265::pMiniSym2-Gus. By contrast, Gus-stained regions of siratro nodules infected by the pMiniSym2-Gus transconjugant were more frequently observed. All nodules formed by NGR234::pXPrpsL426 and those formed by ANU265::pMiniSym2-Gus on *L. leucoephala* were stained throughout infected zones. Scale bars, 1 mm.

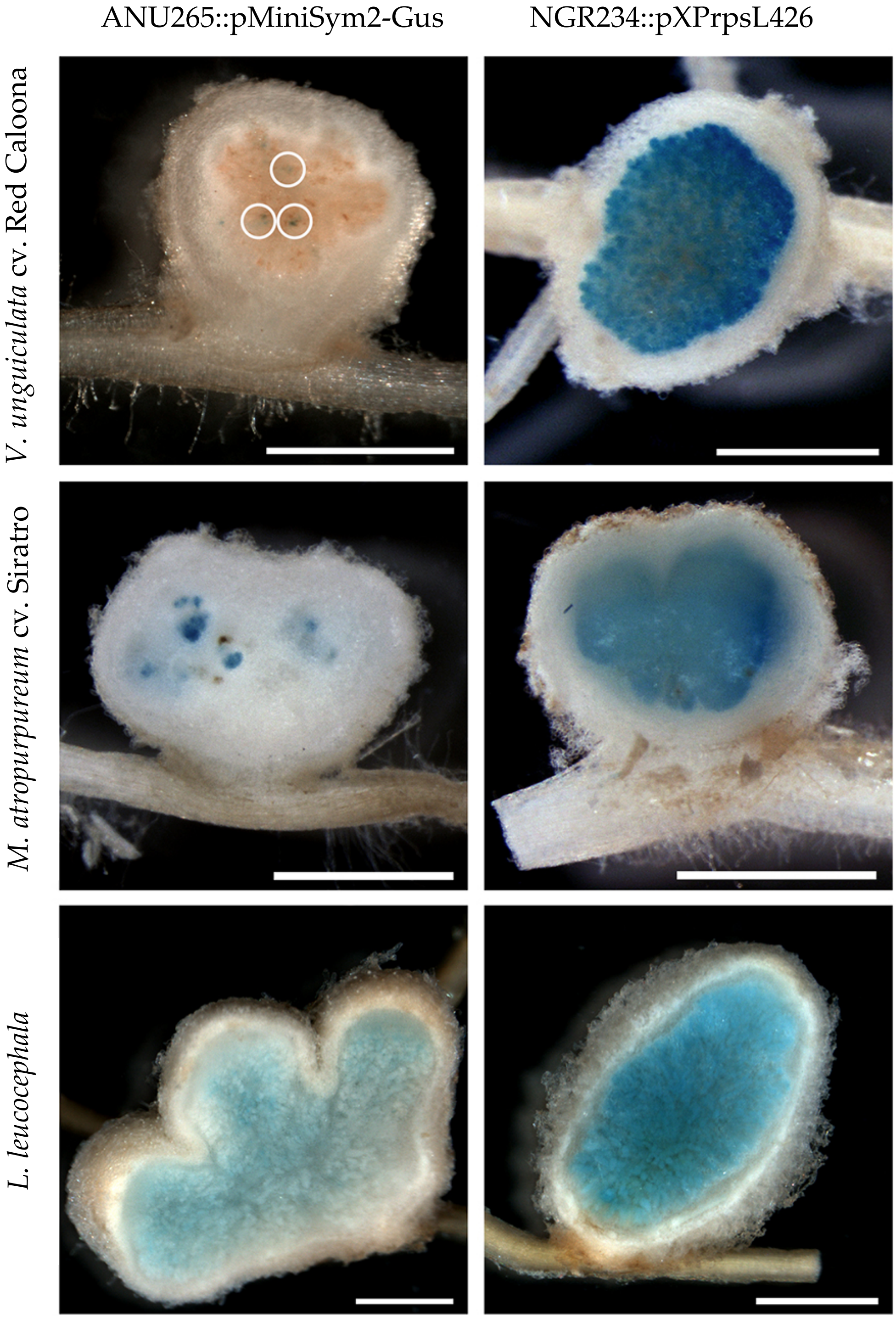

Figure S3. Aborted colonisation of root nodules by ANU265::pMiniSym2 correlates with presence of plant phenolic compounds. Nodules of *Vigna unguiculata* cv. Red Caloona and *Macroptilium atropurpureum* cv. Siratro formed 28 days after inoculation with ANU265::pMiniSym2 or NGR234. 300 µm thick nodule sections were photographed before (**left**) and after staining (**right**) for phenolic compounds. At least six nodules were stained per treatment. Nodule formed by ANU265::pMiniSym2 on cowpea was sectioned across the root vertical axis. Scale bars, 1 mm.

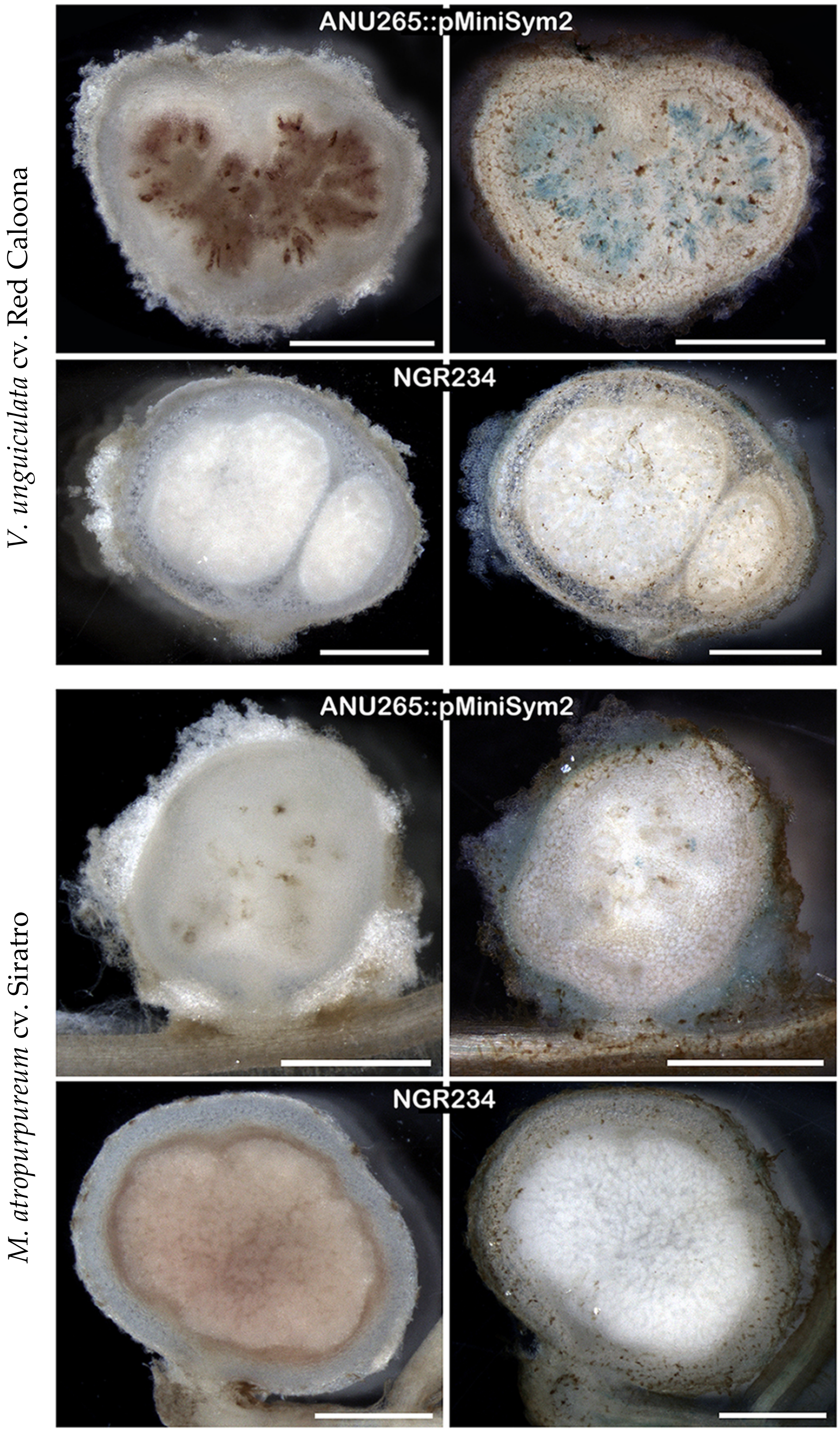

Figure S4. ANU265::pMiniSym2-Gus cannot establish persistent intracellular colonies in nodule cells of *Vigna unguiculata* cv. Red Caloona. Sections of cowpea nodules at 15 dpi with NGR234::pXPrpsL426 (**a to d**) or ANU265::pMiniSym2-Gus (**e** to **h**). Semi-thin sections (1 µm) (panels **a** and **e**) were stained with methylene blue and fuchsine prior to microscopy observation. Details of plant and bacteria cellular ultra-structures can be seen in electron micrographs shown in panels (**b**) to (**d**) and (**f**) to (**h**). White frames in panel (**f**) correspond to the enlarged sections shown in panel (**g**), which shows intracellular bacteria one of which appears to have lost cell integrity and to be degraded (black arrow), and in panel (**h**) where an abnormal infection thread or pocket can be seen. ***bc***, bacteroids; ***cw***, plant cell wall; ***er***, endoplasmic reticulum; ***ib***, intracellular bacteria; ***it***, infection thread; ***it****, abnormal infection thread or infection pocket; ***iz***, infected zone; ***n***, nucleus; ***pbm***, peribacteroid membrane; ***r***, root; ***s***, starch granules; ***v***, vascular bundles. More than six nodules collected on several roots were processed per treatment. Scale bars are 200 µm in (**a**) and (**e**), 5 µm in (**b**) and (**f**), and 2 µm in (**c**, **d**, **g** and **h**).

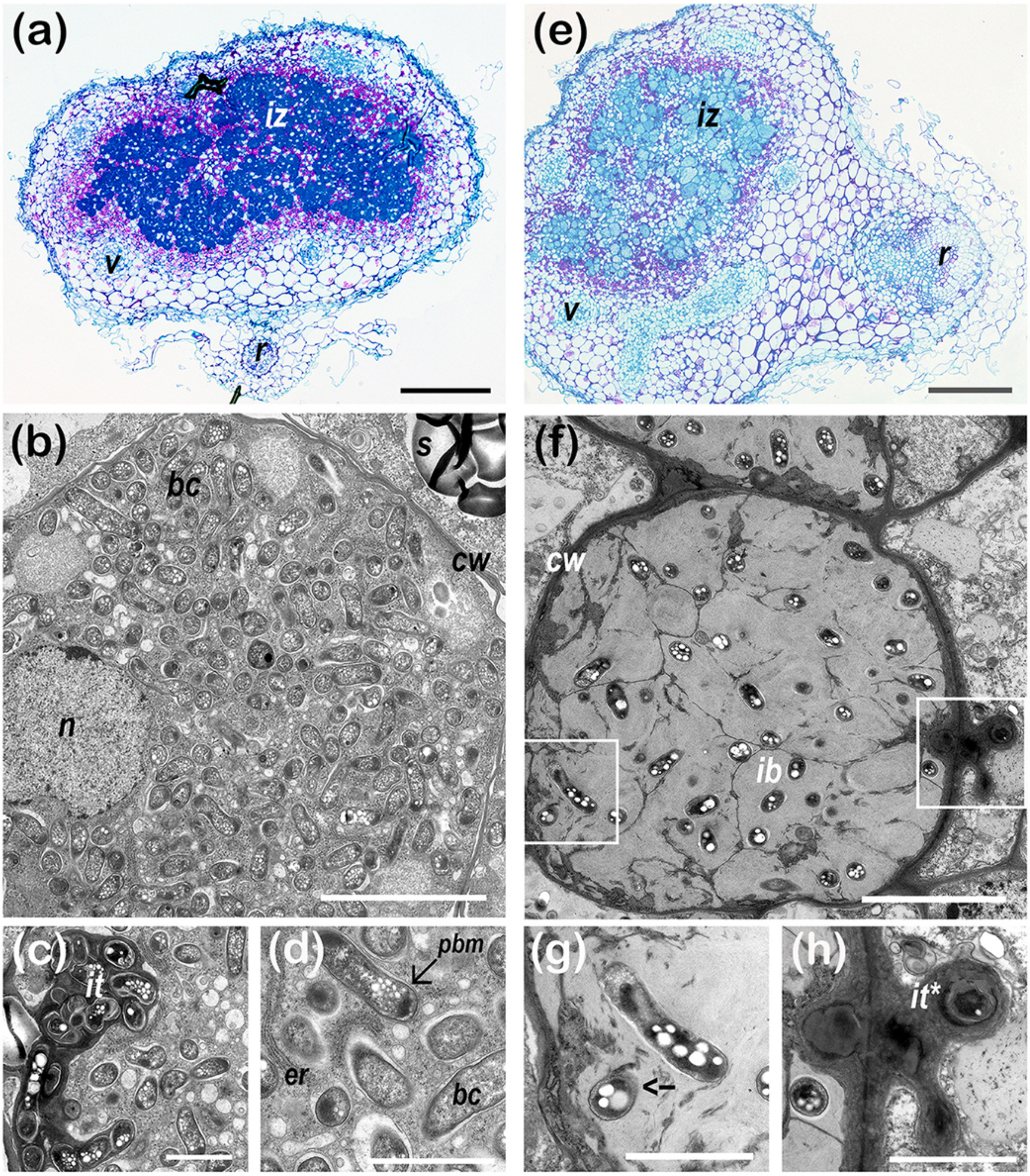

Figure S5. In nodules of *Macroptilium atropurpureum* cv. Siratro, the ANU265::pMiniSym2-Gus transconjugant fails to establish persistent intracellular colonies. At 15 dpi, nodules of siratro formed by NGR234::pXPrpsL426 (panels **a** and **b**) or ANU265::pMiniSym2-Gus (**c** and **d**) were examined by electron microscopy. Micrographs of nodule cells infected by NGR234::pXPrpsL426 (**a** and **b**) show numerous bacteroids that occupy the plant cytoplasm. Intracellular forms of NGR234::pXPrpsL426 are surrounded by peribacteroid membranes, thus forming proficient symbiosome units (panel **b**). By contrast, plant cells infected by ANU265::pMiniSym2-Gus have lost shape and internal cell structures, with several intracellular bacteria being enclosed into vesicles much larger than normal symbiosomes and occasionally showing signs of degradation (**c**). Higher magnification of one necrotic-like lesion with bacteria at various stages of degradation (**d**). ***bc***, bacteroids; ***cw***, plant cell wall; ***er***, endoplasmic reticulum; ***ib***, intracellular bacteria; ***pbm***, peribacteroid membrane; ***s***, starch granules. Scale bars are of 5 (**a** and **c**) and 2 µm (**b** and **d**).

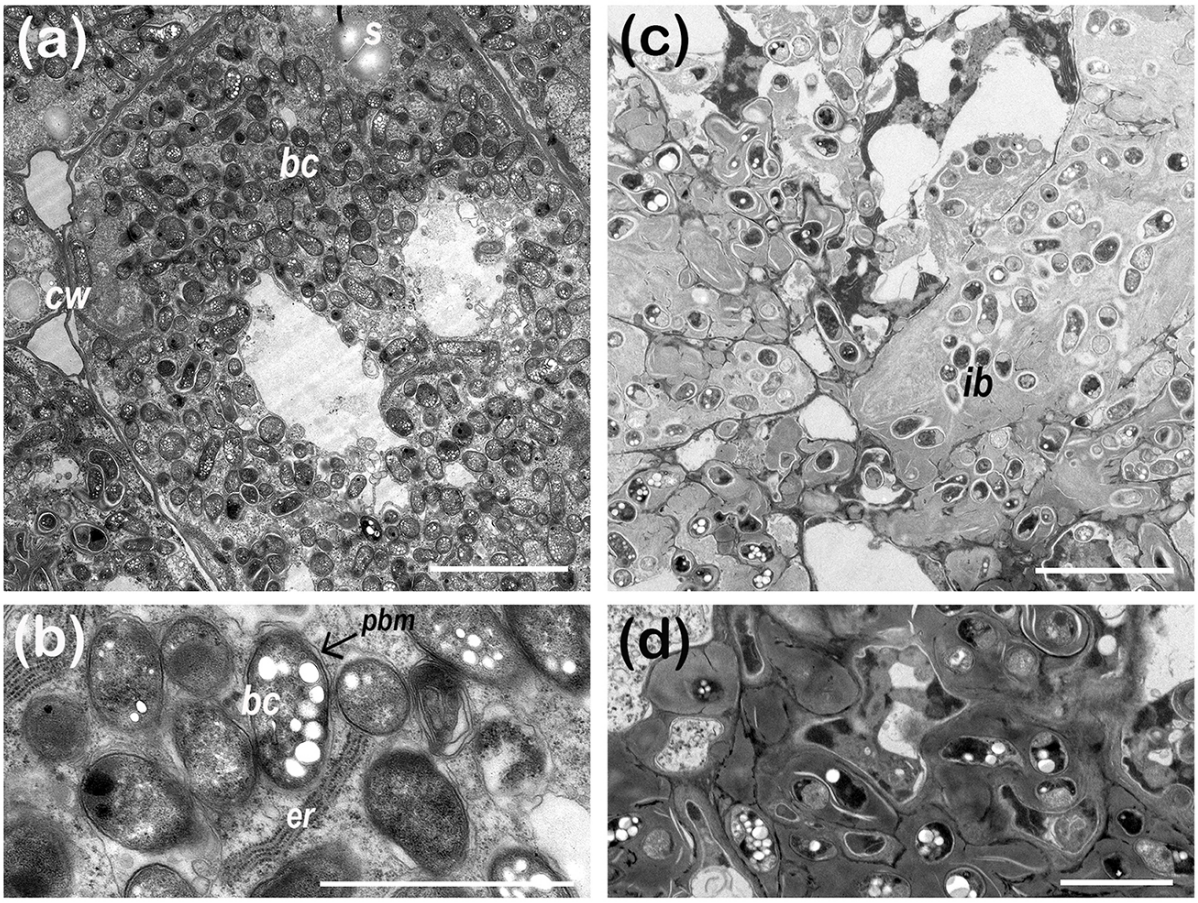

Figure S6. Structures of the Nod-factors produced by NGR234 (**a**) and of those predicted to be secreted by ANU265::pMiniSym2 (**b**). In NGR234, a number of enzymes encoded by the symbiotic plasmid pNGR234a modify the chore structure of Nod-factors (NF) shown in black below (for reviews see Perret et al. 2000 and Brougton et al. 2000). The changes resulting from the activity of these NF-modifying enzymes are highlighted as follows: in dark red for 6-*O*-carbamoylation by NodU; dark blue for 6-*O*-fucosylation by NodZ; light blue for *O*-sulfurylation by NoeE; purple for 2-*O*-methylation by NoeI; light green for *O*-acetylation by NolL; dark green for 3 or 4-*O*-carbamoylation by NolO; and red for *N*-methylation by NodS. The remaining NoeJ (mannose-1-phosphate guanyltransferase), NoeK (phosphomanomutase), NoeL (GDP-mannose 4,6-dehydratase) and NolK (fucose synthase) enzymes are involved in the synthesis of GDP-L-fucose, the substrate for NodZ activity. Except for NodS, all of the NF-modifying enzymes listed above are missing in ANU265::pMiniSym2. Hence, the NF secreted by the pMiniSym2 transconjugant were predicted to be methylated pentamers of *N*-acetylglucosamine that should carry the same fatty acids (C18:1, C18:0 or C16:1) as those found on NF of NGR234.

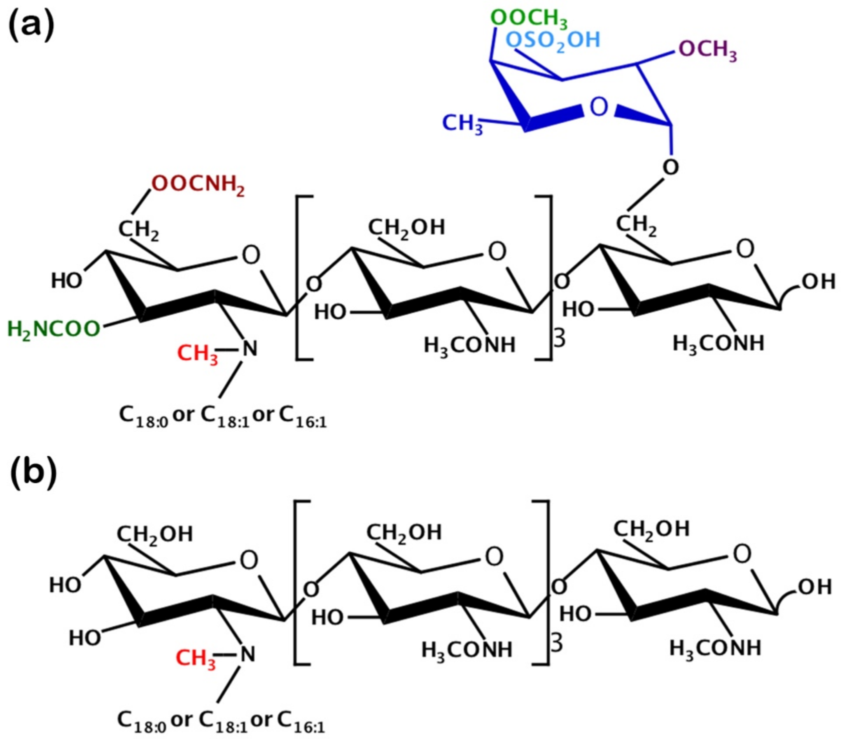

References

Perret, X.; Staehelin, C.; Broughton, W.J. Molecular basis of symbiotic promiscuity. *Microbiol. Mol. Biol. Rev.* **2000**, *64*, 180-201.

Broughton, W.J., Jabbouri, S.; Perret, X. Keys to symbiotic harmony. *J. Bacteriol.* **2000**, *182*, 5641-5652.
